# Program Signaling and Geographic Preferences in the United States Residency Match for Neurosurgery

**DOI:** 10.7759/cureus.69780

**Published:** 2024-09-20

**Authors:** Ahmad Ozair, Jacob T Hanson, Donald K Detchou, Matthew P Blackwell, Abigail Jenkins, Marianne I Tissot, Umaru Barrie, Michael W. McDermott

**Affiliations:** 1 Department of Neurosurgery, University of Maryland School of Medicine, Baltimore, USA; 2 Department of Neurosurgery, Rocky Vista University College of Osteopathic Medicine, Parker, USA; 3 Department of Neurosurgery, University of Pennsylvania Perelman School of Medicine, Pennsylvania, USA; 4 Department of Neurosurgery, Indiana University School of Medicine, Indianapolis, USA; 5 Department of Neurosurgery, University of Texas Southwestern Medical School, Dallas, USA; 6 Department of Neurosurgery, Miami Neuroscience Institute, Baptist Health South Florida, Miami, USA; 7 Division of Neuroscience, Florida International University, Herbert Wertheim College of Medicine, Miami, USA

**Keywords:** clinical training, medical education, neurosurgery residency, nrmp, postgraduate education, postgraduate medical education (pgme), program signal, residency, residency application, residency match

## Abstract

Postgraduate residency training has long been the cornerstone of academic medicine in the United States. The Electronic Residency Application Service (ERAS), managed by the Association of American Medical Colleges (AAMC), is the central residency application platform in the United States for most clinical specialties, with the National Residency Matching Program (NRMP) being the algorithm for matching residency programs with applicants. However, the determination of the best fit between ERAS applicants and programs has been increasingly challenged by the rising number of applicants per residency spot. This application overburdening across competitive specialties led to several adverse downstream effects, which affected all stakeholders. While several changes and proposals were made to rectify the issue of application overburdening, the 2020-2021 ERAS Match Cycle finally saw several competitive specialties, including otolaryngology and urology, utilize a new system of supplemental residency application based on preference signals/tokens. These tokens permit applicants to electronically signal a select number of programs in a specialty of choice, with the program reviewing the application now cognizant that they have been signaled, i.e., the applicant has chosen to use up a limited set of signals for their program. Initial results from otolaryngology and urology, as described in this article, indicated the value of this new system to both applicants and educators. Given the favorable outcomes and broader uptake of the system among other specialties, the field of neurosurgery adopted the utilization of the ERAS-based program signaling and geographic preference for the first time for the 2022-2023 Residency Application Cycle and later opted to continue them for the 2023-2024 and 2024-2025 cycles. For the 2024-2025 Match Cycle, neurosurgery applicants have 25 signals, i.e., a "high-signal" approach, where non-signaled programs have a low interview conversion rate. This literature review discusses the rationale behind the change, the outcomes of other competitive specialties from prior cycles, the evolving nature of the change, and the potential impact on applicants and programs. As we describe in this review, signaling may potentially represent a surrogate form of an application cap. Other considerations relate to cost savings for both applicants and programs from a high-signal approach in neurosurgery. These modifications represent a foundational attempt to alleviate the application overburdening and non-holistic review in the residency application process, including for neurosurgery. While these changes have been a welcomed addition for all stakeholders in residency match cycles so far, further prospectively directed surveys along with qualitative research studies are warranted to better delineate the downstream impact of these changes and guide further optimization of the application system.

## Introduction and background

**Figure 1:**
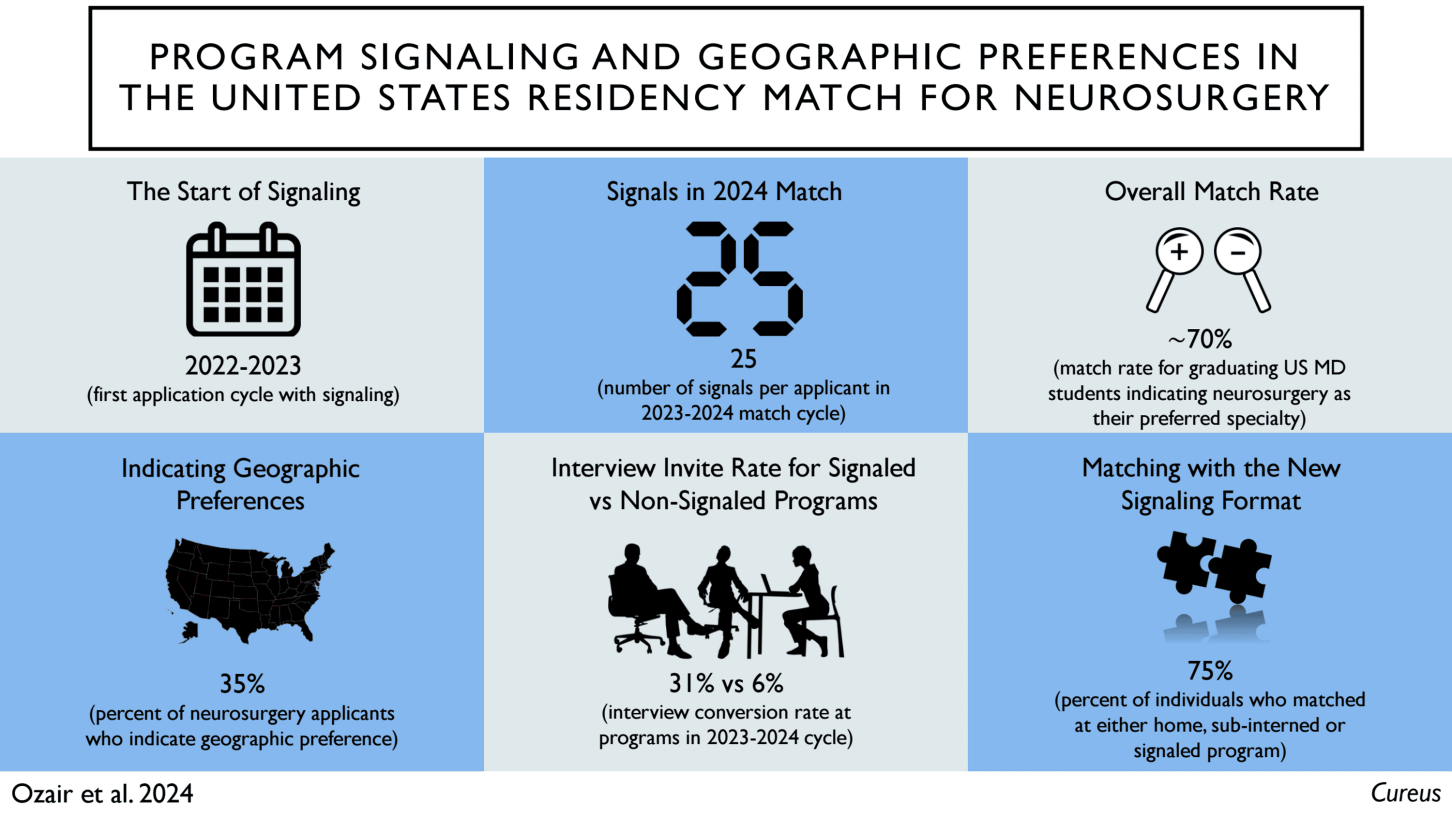
Graphical summary (visual abstract) of the key concepts underlying program signaling for US residency match in neurosurgery Original figure created by the authors for publication based on summary of recent literature.

Postgraduate residency training has long been the cornerstone of academic medicine in the United States, with the country having one of the largest numbers of training positions in the world. The National Resident Matching Program (NRMP) was established in 1952 with over 10,000 positions available in a centralized platform for 6,000 US medical graduates [1,2]. Since then, both the number of positions and applicants have steadily increased. NRMP currently offers more than 38,000 positions for over 50,000 registrants in 2024 through the Electronic Residency Application System (ERAS) [3]. Several competitive specialties, including neurosurgery, have witnessed a considerable increase in applicant interest, with a subsequent rise in applications per program and challenges with a holistic application review. A new paradigm-changing approach of preference signaling was recently instituted, initially by specialties outside of ERAS, and then adopted within ERAS within 2022. The preference signaling system saw rapid adoption within neurosurgery and has become a critical consideration for both applicants and educators. This review discusses the rationale behind this novel system, its subcomponents, stakeholder experiences with outcomes of this new system, the impact of this change on applicants and programs, and future directions (Figure 1).

## Review

Application overburdening and a need for change

The NRMP and later ERAS were created to ensure the best fit between applicants and programs, reduce inequities and undue pressures for early commitment (accomplished via NRMP), and ensure a fair, centralized application platform for all candidates (accomplished via ERAS). The matching algorithm, a generalization of the "stable marriage problem," was first proposed by Gale and Shapley in 1962 and later better described by Roth and Peranson in 1999 [1,2,4]. The algorithm was later implemented in the form of the NRMP Match System ("The Match"). Adopting "The Match" brought substantial benefits for students, but several challenges appeared over the years. Particularly, some specialties saw an excess of applicants, while others had fewer than required to fill all spots in their programs [5].

For neurosurgery, the rising number of applications per candidate has been well-described in the neurosurgical literature, along with reports published by the NRMP and the Association of American Medical Colleges (AAMC). Neurosurgery has historically been in the top five specialties by the average number of ERAS applications submitted per US MD applicant [5]. Data from 2010 to 2020 indicated a match rate of 65% for neurosurgery for all neurosurgery applicants, while graduating US MD applicants typically have a match rate of over 70% [6]. In the 2023 NRMP Match, 78% (211/271) of MD senior applicants and 25% (3/12) of DO senior applicants matched into neurosurgery, with no difference from the following year in MD seniors matching, but slightly lower than DO seniors matching in 2021 (42%). This may be due to an increase in the number of applicants, with more DO students applying into neurosurgery from 2021 to 2022 (14 to 24) versus a smaller increase in MD students applying (269 to 275) from 2021 to 2022, respectively, although there may have been additional factors at play.

However, the rising number of applications from US MD candidates has occurred in the face of a temporally stable proportion of candidates matching to neurosurgery. Pittman described, in 2018, how the match rates of US MD candidates had remained stable for the past 20 years due to a concurrent rise in the number of applicants and neurosurgery training positions [7]. However, the number of programs applied per candidate rose from 40 in 2011 to 65 in 2017. This also coincided with the number of applications received by program directors (PDs). For a program with two spots, the number of applicants received increased steadily from 188 in 2012 to 241 in 2016 (+28%), while the number of interviews offered increased from 35 in 2012 to 40 in 2016 (+14%). Applicants were applying more broadly, accepting more interviews than before, and ranking more programs than before since they perceived other candidates to be doing the same [7].

Programs in competitive specialties, including PDs, faculty, and administrative staff, were particularly overburdened by increasing application numbers. In several specialties, this led to challenges to holistic applicant review, leading to a shift towards the use of filters based on applicants' scores on the US Medical Licensing Examination (USMLE) [8]. Overapplications thus compounded the difficulties faced by educators in determining the best fit between applicant and program. Meanwhile, applicants were also impacted secondary to the use of application filters, lack of holistic review, and, most importantly, the expenditure required to apply to more programs, given that cost per application rose just as rapidly as ERAS application numbers [9]. The recent conversion of the USMLE Step 1 exam and Comprehensive Osteopathic Medical Licensing Examination of the United States (COMLEX-USA) Level I from a numeric score to a Pass/Fail outcome potentially also increased the number of applications per program for competitive specialties [10,11]. The USMLE Step 1 score was previously used to serve as a key indicator for individuals earlier in medical school regarding their competitiveness in the match, while the Step 2 score typically received in the final year of medical school closer to the application deadline presents a unique consideration for use as a personal indicator [11]. The COVID-19 pandemic also accelerated the need for improvements to the residency application process. Historically, applicants applying to neurosurgery residency could indicate their specific interest in a program through sub-internships, advice, advocacy from faculty, in-person interviews, and utilization of departmental networking opportunities. However, with the onset of the COVID-19 pandemic and programs switching to virtual interviews, solidifying and indicating this interest became more difficult. Furthermore, because there was no formal process in place, and due to the variability in access to obtain neurosurgery mentors and attend departmental events, this further contributed to the inequities surrounding the residency application process.

Overapplication-related challenges forced some specialties to attempt innovative strategies for holistic review. Despite the widespread implementation of USMLE Score Filters [11], many of these, to date, proved ineffective, such as the standardized video interview in emergency medicine or the "Secondary Application" in otolaryngology [12,13]. An "interview match," which would come before the "residency match," was also proposed [14]. In 2019, Whipple and colleagues proposed a simulation-based model in otolaryngology where students provided program preferences leading to favorable hypothetical outcomes with holistic application review. Their system sought to make interview invitations more targeted and reduce "application filtering" [15-17]. This model was then implemented by otolaryngology in the 2020-2021 Application Cycle (i.e., the 2021 Match). Here, applicants were provided with five "tokens," also known as "preference signals," to signal their preference for specific programs through the Otolaryngology Program Directors Organization (OPDO) website, notably operating outside the ERAS system. OPDO then provided these signals to each program the day ERAS opened to PDs [18]. Analysis from OPDO reported favorable outcomes for both applicants and programs. Applicants had significantly higher rates of interview invitations from signaled programs (58%) compared to non-signaled programs (14%). Of all otolaryngology stakeholders surveyed, 77% of applicants and 91% of PDs endorsed the continuation of this program [18]. Urology, a specialty that does not participate in ERAS, also reported similar favorable outcomes, with 96% of applicants to a single residency program endorsing the continuation of preference signaling [19]. In response to these challenges and developments, the NRMP released a supplemental application for ERAS for the 2021-2022 Match Cycle, which was utilized first by dermatology, general surgery, and internal medicine programs.

ERAS 2022 and 2023 Supplemental Application: a pilot

In the US residency application match cycles of 2021-2022 (Match 2022) and of 2022-2023 (Match 2023), the supplemental ERAS application was a separate application beyond the standard ERAS packet that aimed to enable students to present more detail about themselves to residency programs, including neurosurgery programs. It also aimed to aid PDs in focusing on applicants that matched the culture and the requirements of their departments [20]. Match 2022 saw three specialties participate in the ERAS Supplemental Application, albeit not across all residency programs in those fields [21]. During this cycle, 87% (117/135) of dermatology programs and 71% of categorical general surgery programs participated in ERAS Supplemental Application. Meanwhile, in internal medicine, 64% (361/566) of categorical track programs and 75% (186/246) of preliminary track programs participated [21]. Interestingly, applicants to these specialties did not uniformly submit supplemental applications, perhaps due to the unfamiliarity with the novel system. Among applicants to each specialty, 93% submitted supplemental applications in dermatology, 87% in categorical general surgery, and 82% in categorical internal medicine, indicating varying levels of engagement with the new system [21].

In Match 2023, 16 specialties utilized preference signaling through ERAS and NRMP, including neurosurgery with eight signals, while ERAS-participating otolaryngology pursued preference signaling via OPDO [22]. The number of preference tokens provided to each residency applicant varied widely among specialties, from three in neurology to 30 in orthopedic surgery (Table 1) [23]. For the 2022-2023 (Match 2023) cycle, the ERAS Supplemental Application opened on August 1, 2022, and closed on September 16, 2022, with data available to programs by late September 2022 [24].

**Table 1:** Specialties that participated in preference signaling and geographic preferences in the 2022-2023 Residency Application Season (Match 2023) Sub-I: sub-internship; ERAS: Electronic Residency Application Service * indicates that home institution is to be signaled unless specifically told to applicants not to. **The table only refers to specialties with program signaling in ERAS and does not include urology and plastic surgery, which had a preference signaling platform outside ERAS. Table reproduced and adapted with permission from the Association of American Medical Colleges (AAMC), with information as of January 1, 2023. Available from: https://students-residents.aamc.org/applying-residencies-eras/about-supplemental-eras-application [23].

Specialty	Indication of meaningful experiences	Indication of geographic preferences	No. of program signals	Signal to home institution	Signal to institutions where Sub-I done	Guidance from specialty societies
Adult neurology	Yes	Yes	3	Yes	Yes	N/A
Anesthesiology	Yes	Yes	5	Yes	Yes	N/A
Dermatology	Yes	Yes	3	No	No	N/A
Diagnostic radiology and interventional radiology	Yes	Yes	6	Yes*	Yes	Yes [25,26]
Emergency medicine	No	No	5	No	No	Yes [27,28]
General surgery	Yes	Yes	5	Yes	Yes	N/A
Internal medicine (categorical)	Yes	Yes	7	No	No	Yes [29]
Internal medicine/psychiatry	Yes	Yes	2	Yes	Yes	N/A
Neurological surgery	Yes	Yes	8	Yes*	Yes	Yes [30,31]
Obstetrics and gynecology	No	No	3 gold; 15 silver	Yes	Yes	Yes [32]
Orthopedic surgery	Yes	Yes	30	Yes	Yes	Yes [33]
Pediatrics	Yes	Yes	5	Yes*	Yes	N/A
Physical medicine and rehabilitation	Yes	Yes	4	Yes	Yes	N/A
Psychiatry	Yes	Yes	5	Yes*	Yes	N/A
General preventive medicine	Yes	Yes	3	Yes	Yes	N/A

In its second year of use in ERAS for Match 2023, and for the first time for neurosurgery, the supplemental application provided a more holistic review of applicants to residency programs [34]. After Match 2022, with 487 applicants applying to more than 72.9 neurological surgery residency programs on average and each program receiving nearly 309 applications on average, it had become increasingly difficult for programs to select the best-fit candidates [35,36]. The addition of the supplemental application was expected to benefit applicants and residency programs alike [34]. The original ERAS Supplemental Application was available at no cost to applicants or programs, and participation for applicants was optional. It was composed of three parts: past experiences, geographic preferences, and preference signaling [21]. For the 2023-2024 Match Cycle, these components of the supplemental application were integrated directly into the ERAS application. Each section potentially provided PDs with a greater opportunity for a more holistic approach to evaluating residency applications.

Past Experiences

This section had applicants describe their (i) up to five meaningful life experiences and (ii) other impactful life experiences. Of the 36% of candidates who responded to an ERAS survey on the new system in Match 2022, more than 90% reported 4-5 meaningful experiences, a trend that continued during the 2023 Match [21,37]. However, less than half of the survey responders (46%) thought the meaningful experiences helped them showcase themselves to programs, a sobering finding. While most PDs considered the "meaningful experiences" section redundant with existing components in the main ERAS application, 80% still utilized it for candidate selection for the 2022 Match. As for impactful life experiences, a significant proportion of dermatology (over 70%), general surgery (almost 50%), and internal medicine (about 35%) PDs found this section provided invaluable context. For the 2023 Match, all specialties excluding emergency medicine and obstetrics and gynecology participated in the past experiences section, with nearly 70% of PDs finding the information from the meaningful and impactful experiences sections helpful and used the responses during the admissions process. 

In neurosurgery, 21% (23/110) of PDs responded to the 2022-2023 Match Cycle survey [38]. Of these programs, 39% (9/23) stated they would use meaningful and impactful experiences when reviewing applications. Of these nine programs, 89% (8/9) stated these were used to holistically review applications and would be used in situations as a tie-breaker between similar candidates and to prepare for the applicant interview. For neurosurgery applicants during the 2022-2023 Match Cycle, 93% (405/435) reported completing the past experiences section, with the vast majority of individuals submitting five experiences [37]. Applicant experiences were categorized by type, with the top experience category in neurosurgery being research at 24%, the highest percentage for across all specialties.

Geographic Preferences

The second component of the erstwhile ERAS Supplemental Application attempted to bring applicants and the programs closer together by having the applicant indicate interest based on geographic region. In this section, applicants provided region-specific details, enabling them to indicate their geographic preference in up to three divisions (regionally grouped states), as well as their preference for urban or rural settings. For the 2022 Match Cycle, the ERAS Supplemental Application had a geographic information section for only dermatology and internal medicine. Of the 36% (9/23) of survey-responding applicants, nearly 67% (6/9) indicated at least one preference for a geographic region [21]. PDs from participating specialties widely considered geographic preferencing useful when deciding between applicants, a trend that continued through to the following cycle. For the 2023 Match Cycle, all specialties participating in the match through ERAS participated in geographic signaling except for emergency medicine and obstetrics and gynecology [37]. While most applicants reported at least one divisional geographic preference, in some of the more competitive specialties, at least 33% of applicants or more did not report any preference. In neurological surgery, 35% of applicants indicated a preference for at least one region, while 63% did not submit a preference. Most of these preferences aligned with the region of applicants' homes or their medical schools. Interestingly, racial/ethnic minority MD/DO candidates and international medical graduate (IMG) applicants had a greater likelihood of not indicating geographic preferences, likely an attempt to avoid getting filtered out by PDs in light of the already lower match results. Of those surveyed, 70-80% of respondents reported submitting a short essay explaining their decision regarding geographic preference [21].

Preference Signaling

The preference signaling system has been the most important aspect of the ERAS Supplemental Application, later integrated into the main ERAS application [15,16]. At its core, preference signaling allows applicants a more meaningful and standardized method to inform PDs and associate program directors (APDs) of their interest in specific residency programs, thus driving holistic review in a system recognized for high-volume applications [21,34]. Preference signaling allows applicants to submit a predetermined number of signals (also referred to as tokens), through ERAS or other designated platforms to programs they are particularly interested in. These signals are sent before interview invitations are extended, providing applicants with the opportunity to draw attention to their preferred programs and emphasize their desire to match with them. While only dermatology, general surgery, and internal medicine participated in preference signaling for the 2022 Match within ERAS, a total of 16 specialties adopted preference signaling integrated within ERAS during the 2023 Match Cycle as well as otolaryngology, urology, and plastic surgery who operated outside ERAS [15,16].

For Match 2022, NRMP reported the first ERAS cycle data available for preference signaling, which was much more granular. Of the 33% of applicants responding to the AAMC survey, 85% of applicants to dermatology, categorical general surgery, and categorical internal medicine had utilized the maximum number of signals available [21]. Meanwhile, nearly a third of preliminary internal medicine applicants had not sent any program signals. Over two-thirds of respondents reported sending signals to residency programs located in the same geographic division as their permanent address and/or medical school.

PDs that responded to the AAMC survey perceived program signals of high value [39]. Over 95% of PDs across specialties in the Match 2022 reported using preference signals during a holistic application review, with >80% reporting using them as a "tie-breaker" for the limited interview invites. Further, nearly 75% of respondent PDs reported that program signals facilitated their identification of candidates they might have overlooked [39].

For Match 2023, neurosurgery applicants were given eight tokens to signal programs of interest, with 96% (110/115) of neurosurgery programs participating in signal preferencing [40]. Of the applicants that signaled, 30% sent a signal to programs in divisions that aligned with their permanent address, while 29% sent a signal to programs that aligned with their home address. Noteworthy, of the 35% of applicants who reported at least one geographic divisional preference, 78% of these individuals sent a signal that aligned with a geographic preference. Therefore, it may be noted by programs that although most applicants do not select a geographic preference, those that do and signal to those programs may be sending a stronger message. This has now been increased to 25 signals in the Match 2024-2025.

Given the recent implementation, literature from neurosurgery remains lacking regarding the supplemental application system. However, academic publications have steadily appeared regarding the experiences with this new system along with its outcomes from use in other specialties. Specifically, during the 2020-2021 Application Cycle, in its first year of use, the ERAS Supplemental Application was adopted by the field of otolaryngology (five preference signals), and a signal was found to increase the likelihood of receiving an interview offer by >250% [18]. Like neurosurgery, otolaryngology is a small field, with 134 residency programs offering 361 residency positions and 574 applicants participating in the 2021 Match process [24]. In Match 2023, the field of neurosurgery allowed signaling to eight programs, and for applicants, consequential decisions had to be made early in the process to identify programs of interest and determine whether these programs are likely to look favorably upon their application. Overall, neurosurgery applicants were and still are expected to benefit from a preference signaling process, as has been expected and/or demonstrated in several other specialties [41-44].

Insights from Match 2024

Based on the value perceived by both educators and applicants, the ERAS Supplemental Application was integrated into the main ERAS application for Match 2024. Preference signaling, therefore, continues for specialties and programs that opt into signaling. For the 2024 Match Cycle, several changes were made to the number of signals available to applicants for each specialty (Table 2). Based on the information from the 2023 Match Cycle, programs such as otolaryngology and neurological surgery increased the number of signals available to applicants to 25, becoming high-signal specialties.

**Table 2:** Preference signals available for each specialty by year Table reproduced and adapted with permission from the Association of American Medical Colleges (AAMC), with information as of March 1, 2024. Available from: https://students-residents.aamc.org/applying-residencies-eras/about-supplemental-eras-application, https://students-residents.aamc.org/applying-residencies-eras/myeras-application-and-program-signaling-2023-24, and https://www.aamc.org/media/64591/download [23].

Specialty	Match 2022	Match 2023	Match 2024
Anesthesiology	-	-	5 (gold), 10 (silver)
Child neurology and neurodevelopmental disabilities	-	-	3
Dermatology	3	3	3 (gold), 25 (silver)
Diagnostic radiology and interventional radiology	-	6	6 (gold), 6 (silver)
Emergency medicine	-	5	7
Family medicine	-	-	5
General surgery	5	5	5
Internal medicine	5	7	7
Internal medicine and psychiatry	-	2	2
Neurological surgery	-	8	25
Neurology	-	3	3
Obstetrics and gynecology	-	3 (gold), 15 (silver)	3 (gold), 15 (silver)
Orthopedic surgery	-	30	30
Otolaryngology	4	7	25
Pathology	-	-	5
Pediatrics	-	5	5
Physical medicine and rehabilitation	-	4	5
Public health and general preventive medicine	-	3	3
Psychiatry	-	5	5
Thoracic surgery	-	-	3

Neurosurgery's transition to a high-signal specialty has had several effects, as seen in Table 3. These include changes in the interview conversion rate, which refers to the percentage of applicants who receive interviews after submitting their residency applications. In the 2024 Match Cycle, the signal-to-interview conversion rate remained consistent at 31% (range 22-48%), comparable to 2023 [45]. Interestingly, during the 2022-2023 cycle, applicants who did not signal a program had an interview conversion rate of 16%. However, in the subsequent 2023-2024 cycle, programs that were not signaled converted to an interview only 6% of the time. This decrease is consistent with trends observed in other high-signal programs such as orthopedic surgery (30 signals with a non-signal conversion rate of 1%) and otolaryngology (25 signals with a non-signal conversion rate of 2%). Similar to otolaryngology, neurosurgery also now is utilizing 25 signals, and the interview conversion rate of non-signaled applications is expected to drop further.

**Table 3:** Total number of applicants along with the number of applications per applicant and per program across recent match cycles in neurosurgery ERAS: Electronic Residency Application Service; DO: doctor of osteopathic medicine; IMG: international medical graduate; MD: doctor of medicine Table reproduced and adapted with permission from the Association of American Medical Colleges (AAMC), with information as of March 1, 2024. Available from: https://www.aamc.org/data-reports/data/eras-statistics-data [23,46].

Grad type	ERAS 2019	ERAS 2020	ERAS 2021	ERAS 2022	ERAS 2023	ERAS 2024
Number of applicants						
DO	30	25	23	29	21	26
IMG	101	124	147	121	108	117
MD	306	336	330	334	331	356
Overall	437	485	500	484	460	499
Number of applications per applicant						
DO	44.77	49.84	80.61	71.69	58.62	69.35
IMG	50.62	55.73	59.32	55.11	59.08	70.52
MD	68.18	71.38	79.87	79.83	78.02	70.25
Overall	62.51	66.26	73.86	73.16	72.69	70.27
Number of applications per program						
DO	11.99	10.93	16.12	18.24	10.70	15.54
IMG	45.65	60.61	75.83	58.49	55.49	71.13
MD	186.27	210.37	229.19	233.89	224.57	215.60
Overall	243.91	281.91	321.14	310.62	290.76	302.28

While neurosurgery maintained its trends in geographic preferences, significant shifts were observed in orthopedic surgery and otolaryngology for the 2024 Match. Notably, in the past, neurosurgery was the sole specialty where the majority of applicants did not select at least one divisional preference (63% for the 2023 Match) [47]. However, for the 2024 Match, the majority of applicants across neurosurgery, otolaryngology, and orthopedic surgery opted not to indicate any geographic preference. This shift may be attributed to strategic considerations by applicants aiming to optimize their chances of matching with their preferred programs. With the increased number of signals available for high-signaling specialties, applicants may prioritize aligning their program signals with their geographic preferences, if any, to maximize their likelihood of securing interviews and matching successfully. By choosing not to indicate a geographic preference, applicants can ensure that their program signals are not restricted by geographic constraints or conflicts, potentially increasing their flexibility in program selection and enhancing their overall competitiveness in the match process. While it remains unclear if these shifts are directly related to the results from the 2022-2023 cycle, future cycles may provide more insight.

Further considerations

Given its continued use, there exist several considerations concerning preference signaling for applicants and programs alike [41-44,48]. Firstly, it is evident that, based on both outcomes of and experiences with preference signaling, the system is likely to stay. More than half of the respondents in the AAMC survey administered for Match 2022 felt that these signals may help applicants get noticed, even though 2022 was the first ERAS implementation of this novel system. Carpinito et al., in 2023, reported, through a survey of urology residency applicants, that >80% felt the signaling system should continue in the future [49]. Of the respondents, two-thirds matched to a urology program that they signaled to or had done a rotation at, further demonstrating utility to applicants [49]. Kim et al. reported that among urology residency applicants, a survey with a 24% response rate indicated that three-fourths of candidates matched at places that were either (1) home programs, (2) away rotation programs, or (3) signaled programs [50]. Leopold and colleagues, surveying all applicants to a single New Jersey institution, reported that >95% of respondents recommended continuation of program signaling [19]. Traxel and colleagues reported from urology that the interview invite rate in Urology Match 2022 was merely 10% at a non-signaled program and 51% at a signaled program [51]. Pletcher et al. surveyed otolaryngology applicants utilizing preference signaling through AAMC-OPDO collaboration for the 2021 Match Cycle. They reported in 2023 that the median interview selection rate at a signaled program for an applicant was 48% (95% CI: 27-68%), while the invite rate at a non-signaled residency program was 10% (95% CI: 7-13%) [18]. Meanwhile, Grauer et al. recently reported data on all applicants and programs in urology, demonstrating that a program signal was the most significant variable associated with a candidate's reception of an interview invite [52]. 

Given these data, the concurrent presence of a program signal is rapidly becoming a screening tool ("filter") for programs to focus on the careful review of only those applicants who consider the program as their top 25, i.e., a "soft cap" for applications in a high-signal specialty [44,52-55]. Some programs meanwhile are using Step 2 score thresholds, replacing the previously used Step 1 score, as an additional tool for filtering applicants [11].

Meanwhile, recent research by Standiford and colleagues highlighted the impact of preference signaling on the distribution of interview invites among residency applicants. Their study utilized self-reported applicant survey data to divide candidates into four quartiles based on the overall interview offer rates ("self-reported number of interviews/self-reported number of applications submitted"), modeling a distribution of interview invites [53]. For the 2023 cycle, they reported that top quartile candidates received fewer invites, while the candidates in the other quartiles experienced an increase. Thus, this shift suggests that preference signaling led to a more equitable distribution of interviews across the candidate pool, which may potentially improve equity and inclusion, a key consideration in neurosurgery today. Additionally, the changes to the ERAS application may also provide more equitable opportunities for students without neurosurgery programs at their home institutions. By enhancing the visibility of these students to residency PDs, their applications may be selected and holistically reviewed, ultimately improving their chances of securing interviews. Finally, these new additions may also increase the diversity of neurosurgery applicants to include more DO and IMG students at smaller programs who may not have historically matched a student from these applicant pools.

Preference signaling carries unique issues related to equity in the residency match. Smaller neurosurgery residency programs that may not be as well-known may not receive as many signals from applicants as more renowned programs. In ERAS 2022 Supplemental Application findings, a quarter of programs in each specialty received nearly half the signals [21]. This unsurprising point indicates that for certain programs, the value of the signal may be minimized due to over-signaling and the value of a signal may vary between programs. However, this could be seen as a benefit that when an applicant signals a smaller program, it means with an extremely high likelihood that a person would like to train there. Additionally, PDs might be more hesitant to send out interviews to applicants who may seem like a great fit for the program but don't end up signaling them, further exacerbating unconscious bias within the application process. In contrast, applicants might also over-strategize their program selections. For instance, they may believe that being a well-rounded applicant or having completed rotations at a particular program increases their likelihood of receiving an interview invitation, regardless of their signal preference. Consequently, they may choose to signal a different program, one they may not feel as confident about securing an interview at. However, over 70% of respondents in the AAMC survey discussed above reported that their program signals were reflective of their true preferences at the time of application [21]. Another potential challenge for applicants to effectively utilize preference signaling could arise from the timing of submission. Because signals are submitted alongside the ERAS application early in the cycle, applicants' interests, whether in a specific program or a broader category of neurosurgery programs (e.g., academic versus community), may evolve over the course of the interview season, with no opportunity available to adjust their signal preferences accordingly. To combat this, residency programs need to provide historical data on successful applicants matching into their respective programs and what unique aspects the program has to offer to applicants.

Specific factors behind signaling a specific program in neurosurgery remain unknown and warrant further research. Feroe et al., in 2023, introduced the concept of a "strategic signaling spear" for orthopedic surgery residency applications (i.e., a framework to conceptualize the appropriate use of preference signaling) [48]. In the AAMC 2022 survey, the top three factors reported by candidates (applying to dermatology, general surgery, or internal medicine) when considering signaling were the concordance between their interests and program strengths (67%), geographic preference (66%), and quality of clinical training (54%) [21]. Kim et al. surveyed applicants for the 2022 Urology Residency Match for factors affecting their decision-making. They found that 73% of signals were primarily influenced by program location, while nearly half were influenced by reputation. Applicants reported sending a third of signals to perceived "target" urology residency programs, a third to "reach" programs, and 8% to "safety" programs [50]. 

However, certain questions and considerations remain unclear. Aspiring neurosurgery trainees continue to have questions regarding signaling to their home program, if applicable, or to programs where they completed a neurosurgery sub-internship, when applicable. Guidance from the SNS has been helpful, although it must be noted that this guidance has not been uniformly followed across programs [30,31]. Signaling may potentially represent a surrogate form of an application cap. At certain large programs receiving a high volume of signals, PDs may only offer interview invitations to applicants who have indicated a preference for their training program. This is reinforced by the Match 2024 data for high-signal specialties. For neurosurgery, programs that did not receive a signal from an applicant only converted to an interview 6% of the time, with even lower conversion rates for orthopedic surgery (1%) and otolaryngology (2%) [45].

For neurosurgery, one of the primary purposes of signaling was to add an element to the process to allow for programs to provide a more holistic review of applicants and reduce the overall application burden, relating to the artificial application cap. With that in mind, the average number of applications per neurosurgery applicant only decreased by two absolute percentage points overall from 2023 to 2024 [54,55]. In contrast, orthopedic surgery saw a 16% decrease in the average number of applications per applicant, while otolaryngology saw a 25% decrease. Orthopedic surgery has had two cycles now as a high-signaling specialty, and with only 1% of non-signaled applications converting to an interview, applicants must consider this factor when applying above the signaling cap. When discussing the pros and cons of the three types of approaches to program signaling, the AAMC explicitly states one of the pros for large signal specialties is that "applicants may more seriously consider applying to programs they don't signal" [46,54]. Whether an additional drop will be observed in the number of neurosurgery applications submitted per applicant awaits to be seen. Other considerations relate to cost savings for both applicants and programs from a high-signal approach in neurosurgery, a consideration that has been recently described in otolaryngology [56].

Given that 2022-2023 and 2023-2024 have been the first two match cycles utilizing preference signaling in neurosurgery, there may be additional unforeseen benefits and challenges for both applicants and programs that remain to be reported. Work is needed in neurosurgery to clarify the distribution of applicant signals across signaling highly competitive ("reach") programs and less competitive ("safety") programs, as has been recently reported in radiology [57]. Further studies should examine the impact of preference signaling on the interview conversion rate of neurosurgery applicants at differentially ranked programs (Blue Ridge Institute of Medical Research NIH Funding, Doximity, US News and World Reports, or other ranking approaches), hidden additional challenges faced by racial and ethnic minority candidates, role of signaling in applicants with high vs. low USMLE Step 2 scores, and how the approach indirectly provides for an application cap.

## Conclusions

The modifications to the ERAS application, in particular the addition of geographic and program preference signaling, represent a major multi-specialty effort to alleviate the application overburdening and non-holistic review in the residency application process, including for neurosurgery. While these changes have been a welcomed addition for all stakeholders in residency match cycles so far, further prospectively directed efforts that minimize well-recognized challenges of survey-based approaches are warranted. 
